# Supplementary material for: Hematodinium sp. infection does not drive collateral disease contraction in a crustacean host
Source: eLife. 2022 Feb 18;11:e70356. doi: 10.7554/eLife.70356 (PMC8856654; doi:10.7554/eLife.70356)
Supplement: Supplementary file 1. — Linear regression of Hematodinium intensity in the liquid tissue (parasites per mL haemolymph) log transformed [Y = log(y + 1)] (liquid tissues) against average histology severity rating from gills and hepatopancreas (solid tissues) for infection severity (n = 108). [file elife-70356-supp1.docx]

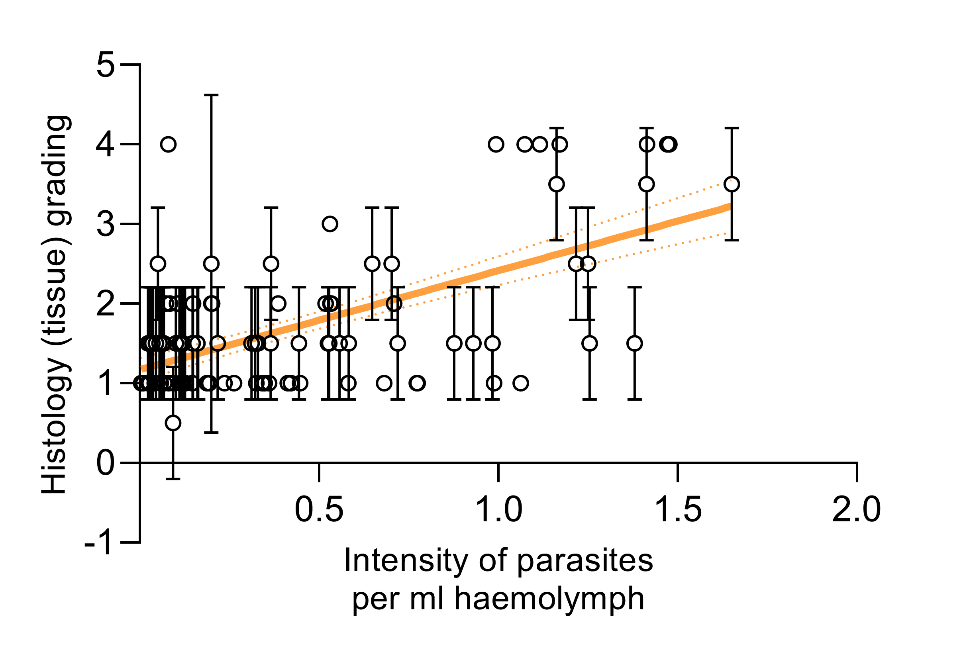


Linear regression of *Hematodinium* intensity in the liquid tissue (parasites per mL haemolymph) log transformed [Y=log(y+1)] (liquid tissues) against average histology severity rating from gills and hepatopancreas (solid tissues) for infection severity (*n* = 108). Histology slides were graded according to Smith et al (2015). Values represent mean + 95% CI.
