# Supplementary material for: Hematodinium sp. infection does not drive collateral disease contraction in a crustacean host
Source: eLife. 2022 Feb 18;11:e70356. doi: 10.7554/eLife.70356 (PMC8856654; doi:10.7554/eLife.70356)
Supplement: Supplementary file 2. [file elife-70356-supp2.docx]

**Supplementary file 2 - table 1.** Full model used to predict response variable of presence of one or more coinfections before reduction. Asterisk denotes significance (*P* ≤ 0.05).

| **Model** | **Predictor variable** | **Estimate (slope)** | **SE** | ***P*-value** |
| --- | --- | --- | --- | --- |
| **Model S1** | | | | |
| CoInfect1 ~ Hemat + | Hemat | 0.07426 | 0.27317 | 0.78575 |
| Location + Season + CW + | Location (pier) | -0.35599 | 0.31114 | 0.25256 |
| Sex + Colour + LimbLoss | Season (Spring) | -0.15239 | 0.41978 | 0.71659 |
| + Fouling | Season (Summer) | -0.03200 | 0.42583 | 0.94010 |
|  | Season (Winter) | -0.49015 | 0.46174 | 0.28845 |
| *df* = 312 | CW | -0.04199 | 0.01542 | 0.00646 ** |
| AIC: 352.76 | Sex (male) | 0.38302 | 0.33016 | 0.24601 |
|  | Colour (orange) | 0.01962 | 0.39744 | 0.96062 |
|  | Colour (yellow) | 0.31485 | 0.34121 | 0.35614 |
|  | Limb loss | -0.56282 | 0.34125 | 0.09909 |
|  | Fouling | -0.62346 | 0.43881 | 0.15538 |
|  |  |  |  |  |
| **Model S2** |  |  |  |  |
| CoInfect1HEMAT ~ | Location (pier) | -0.63614 | 0.46889 | 0.1749 |
| Location + Season + CW + | Season (Spring) | -0.23071 | 0.66875 | 0.7301 |
| Sex + Colour + LimbLoss | Season (Summer) | -1.02654 | 0.67776 | 0.1299 |
| + Fouling | Season (Winter) | -0.91081 | 0.71682 | 0.2039 |
|  | CW | -0.04612 | 0.02204 | 0.0364 * |
| *df* = 154 | Sex (male) | -0.09198 | 0.48664 | 0.8501 |
| AIC: 170.73 | Colour (orange) | -1.22687 | 0.76605 | 0.1093 |
|  | Colour (yellow) | 0.42058 | 0.51301 | 0.4123 |
|  | Limb loss | -1.42522 | 0.59542 | 0.0167 * |
|  | Fouling | -0.96667 | 0.70577 | 0.1708 |
|  |  |  |  |  |
| **Model S3** |  |  |  |  |
| CoInfect1CONTROL ~ | Location (pier) | 0.03996 | 0.45151 | 0.929 |
| Location + Season + CW + | Season (Spring) | -0.21535 | 0.65644 | 0.743 |
| Sex + Colour + LimbLoss | Season (Summer) | 0.57898 | 0.62695 | 0.356 |
| + Fouling | Season (Winter) | -0.41023 | 0.70226 | 0.559 |
|  | CW | -0.03354 | 0.02244 | 0.135 |
| *df* = 157 | Sex (male) | 0.80267 | 0.48811 | 0.100 |
| AIC: 183.17 | Colour (orange) | 0.67402 | 0.52790 | 0.202 |
|  | Colour (yellow) | 0.13042 | 0.54066 | 0.809 |
|  | Limbloss | -0.04315 | 0.46013 | 0.925 |
|  | Fouling | -0.63988 | 0.62217 | 0.304 |
|  |  |  |  |  |
| **Model S4** | | | | |
| Hemat ~ Sacc + | Sacculina | 0.22986 | 0.35032 | 0.512 |
| Trematodes + Haplo + | Trematodes | -0.19201 | 0.40809 | 0.638 |
| Microsp + Vibrio + | Haplosporidia | -0.34915 | 0.92242 | 0.705 |
| Fungi | Microsporidia | 12.46745 | 535.41241 | 0.981 |
|  | Vibrio | 1.11717 | 1.16173 | 0.336 |
| *df* = 322 | Fungi | 0.01855 | 1.00811 | 0.985 |
| AIC: 458.4 |  |  |  |  |
|  | | | | |
| **Model S5** | | | | |
| HematDock ~ Sacc + | Sacculina | 0.28768 | 0.37016 | 0.437 |
| Trematodes + Haplo + | Trematodes | NA | NA | NA |
| Microsp + Vibrio + | Haplosporidia | NA | NA | NA |
| Fungi | Microsporidia | 13.87292 | 882.74422 | 0.987 |
|  | Vibrio | 0.76952 | 1.23717 | 0.534 |
| *df* = 172 | Fungi | NA | NA | NA |
| AIC: 245.49 |  |  |  |  |
|  |  |  |  |  |
| **Model S6** |  |  |  |  |
| HematPier ~ Sacc + | Sacculina | NA | NA | NA |
| Trematodes + Haplo + | Trematodes | -0.25615 | 0.42913 | 0.551 |
| Microsp + Vibrio + | Haplosporidia | -0.40284 | 0.92952 | 0.665 |
| Fungi | Microsporidia | NA | NA | NA |
|  | Vibrio | 14.51863 | 882.74339 | 0.987 |
| *df* = 149 | Fungi | -0.04744 | 1.01729 | 0.963 |
| AIC: 216.00 |  |  |  |  |
|  |  |  |  |  |

*Statistically significant **P* ≤ 0.05, ***P* ≤ 0.01, ****P* ≤ 0.001

*Abbreviation*: SE, standard error

**Supplementary file 2 - table 2.** Full models used to predict response variable of presence of one or more coinfections, using only those animals with *Hematodinium* sp. at a **clinical level** (i.e., via blood smear) before reduction. Asterisk denotes significance (*P* ≤ 0.05).

| **Model** | **Predictor variable** | **Estimate (slope)** | **SE** | ***P*-value** |  |
| --- | --- | --- | --- | --- | --- |
| **Model S7** | | | | | |
| CoInfect1~HematSmear | HematSmear | 0.01288 | 0.30756 | 0.9666 |  |
| + Location + Season | Location (Pier) | -0.19192 | 0.34440 | 0.5774 |  |
| + CW + Sex + Colour | Season (Spring) | -0.23386 | 0.45589 | 0.6080 |  |
| + LimbLoss + Fouling | Season (Summer) | 0.18027 | 0.45682 | 0.6931 |  |
|  | Season (Winter) | -0.30667 | 0.50302 | 0.5421 |  |
| *df*=262 | CW | -0.03384 | 0.01685 | 0.0446 * |  |
| AIC: 297.43 | Sex(male) | 0.63119 | 0.36627 | 0.0848 . |  |
|  | Colour(orange) | 0.09963 | 0.42261 | 0.8136 |  |
|  | Colour(yellow) | 0.38440 | 0.36982 | 0.2986 |  |
|  | Limbloss | -0.47313 | 0.36416 | 0.1939 |  |
|  | Fouling | -0.66152 | 0.47311 | 0.1620 |  |
|  |  |  |  |  |  |
| **Model S8** |  |  |  |  |  |
| CoInfectHematSmear~ | Location(pier) | -0.33089 | 0.60864 | 0.5867 |  |
| Location + Season + CW + | Season(Spring) | -0.74175 | 0.85578 | 0.3861 |  |
| Sex + Colour + LimbLoss | Season(Summer) | -1.08805 | 0.86270 | 0.2072 |  |
| + Fouling | Season(Winter) | -0.70131 | 0.90496 | 0.4384 |  |
|  | CW | -0.02862 | 0.02739 | 0.2961 |  |
| *df*=104 | Sex(male) | 0.53527 | 0.60665 | 0.3776 |  |
| AIC:117.19 | Colour(orange) | -2.10697 | 1.23133 | 0.0871 |  |
|  | Colour(yellow) | 0.53774 | 0.60687 | 0.3756 |  |
|  | Limbloss | -1.34844 | 0.70417 | 0.0555 |  |
|  | Fouling | -1.20895 | 0.89595 | 0.1772 |  |
|  |  |  |  |  |  |
| **Model S9** |  |  |  |  |  |
| HematSmear~Sacc + | Sacculina | 9.353e-02 | 1.342e-01 | 0.796 |  |
| Trematodes + Haplo + | Trematodes | -3.466e-03 | 3.621e-01 | 0.994 |  |
| Microsp + Vibrio + | Haplosporidia | -1.593e+01 | 4.335e-01 | 0.988 |  |
| Fungi | Microsporidia | 1.657e+01 | 1.073e+03 | 0.994 |  |
|  | Vibrio | 6.325e-01 | 2.400e+03 | 0.531 |  |
| *df*=322 | Fungi | -1.593e+01 | 1.009e+00 | 0.989 |  |
| AIC: 419.41 |  |  |  |  |  |
|  |  |  |  |  |  |
| **Model S10** |  |  |  |  |  |
| HematDockSmear~Sacc+ | Sacculina | 0.10869 | 0.284 | 0.776768 |  |
| Trematodes+Haplo+ | Trematodes | NA | NA | NA |  |
| Microsp+Vibrio+ | Haplosporidia | NA | NA | NA |  |
| Fungi | Microsporidia | 15.25921 | 882.74422 | 0.986208 |  |
|  | Vibrio | -0.04546 | 1.23849 | 0.970718 |  |
| *df*=172 | Fungi | NA | NA | NA |  |
| AIC: 230.39 |  |  |  |  |  |
|  |  |  |  |  |  |
| **Model S11** |  |  |  |  |  |
| HematPierSmear~Sacc+ | Sacculina | NA | NA | NA |  |
| Trematodes+Haplo+ | Trematodes | -0.0208 | 0.4566 | 0.96366 |  |
| Microsp+Vibrio+ | Haplosporidia | -16.9467 | 1769.2356 | 0.99236 |  |
| Fungi | Microsporidia | NA | NA | NA |  |
|  | Vibrio | 18.1812 | 3956.1803 | 0.99633 |  |
| *df*= 149 | Fungi | -16.9509 | 1978.0902 | 0.99316 |  |
| AIC: 191.28 |  |  |  |  |  |
|  |  |  |  |  |  |
| **Model S12** |  |  |  |  |  |
| CoInfect1 (overall)  ~ HematIntensity | HematIntensity | 0.006590 | 0.006835 | 0.335 |  |
|  |  |  |  |  |  |
| *df*= 316 |  |  |  |  |  |
| AIC: 346.12 |  |  |  |  |  |
|  |  |  |  |  |  |
| **Model S13** |  |  |  |  |  |
| CoInfect1Pier ~HematIntensity | HematIntensity | 0.006624 | 0.006834 | 0.332 |  |
|  |  |  |  |  |  |
| *df*= 145 |  |  |  |  |  |
| AIC: 157.62 |  |  |  |  |  |
|  |  |  |  |  |  |
| **Model S14** |  |  |  |  |  |
| CoInfect1Dock~ HematIntensity | HematIntensity | 0.009928 | 0.044064 | 0.822 |  |
|  |  |  |  |  |  |
| *df*= 170 |  |  |  |  |  |
| AIC: 192.33 |  |  |  |  |  |

*Statistically significant **P*≤0.05, ***P*≤0.01, ****P*≤0.001

*Abbreviation*: SE, standard error
