# Supplementary material for: Hematodinium sp. infection does not drive collateral disease contraction in a crustacean host
Source: eLife. 2022 Feb 18;11:e70356. doi: 10.7554/eLife.70356 (PMC8856654; doi:10.7554/eLife.70356)
Supplement: Supplementary file 3. [file elife-70356-supp3.docx]

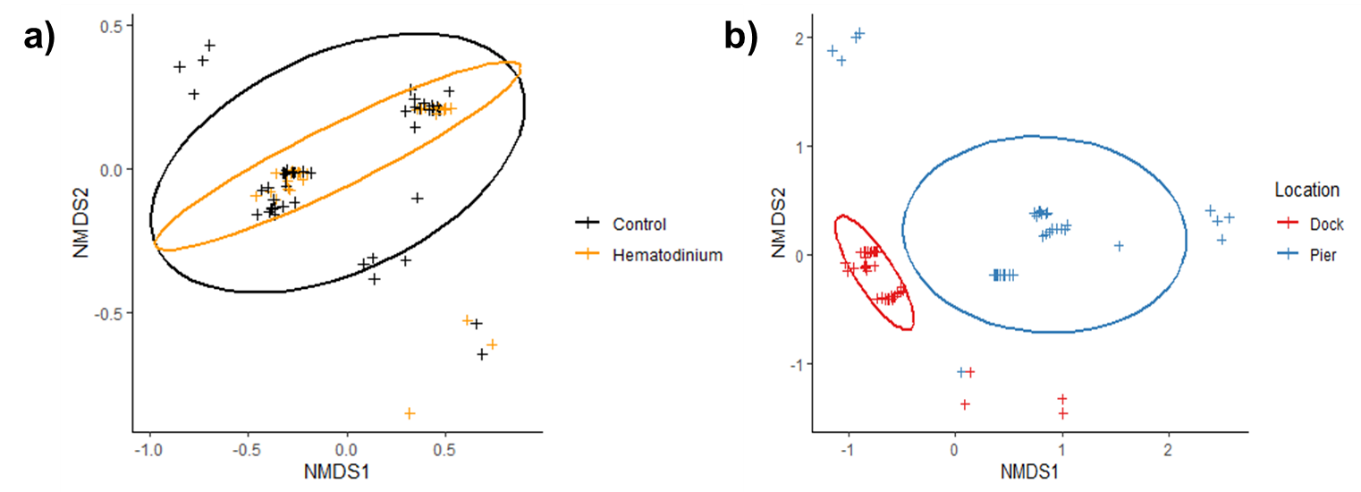


**nMDS ordinations of parasite (co-infection) community structure** excluding the crabs that were positive for PCR alone (i.e., subclinical infections). Data is derived from crabs assessed for *Hematodinium* using haemolymph inspection via phase contrast microscopy, gill/hepatopancreas using histology, and PCR (a). Non-metric multidimensional (nMDS) ordination co-infection/parasite (haplosporidia, microsporidia, *Vibrio* spp., fungal species, *Sacculina* *carcini* and trematodes) community structure in crabs that were *Hematodinium* sp. positive (orange) and *Hematodinium* sp. free (black – control). (b). Non-metric multidimensional (nMDS) ordination co-infection/parasite (haplosporidia, microsporidia, *Vibrio* spp., fungal species, *Sacculina carcini* and trematodes) community structure in crabs from Dock (red) and Pier (blue) locations. Analyses were done using square-root transformation of species’ abundances and Bray-Curtis similarity. Each point denotes an individual crab with one or more co-infections.
