# Supplementary material for: Hematodinium sp. infection does not drive collateral disease contraction in a crustacean host
Source: eLife. 2022 Feb 18;11:e70356. doi: 10.7554/eLife.70356 (PMC8856654; doi:10.7554/eLife.70356)
Supplement: Supplementary file 4. [file elife-70356-supp4.docx]

**Supplementary File 4.** Blast results and accession numbers of positive samples obtained in this study and used for phylogenetic analyses.

| **GenBank ID** | **Sample** | **Target** | **Primers** | **Sample type** | **Top blast** | **Query cover** | **% ID** |
| --- | --- | --- | --- | --- | --- | --- | --- |
| MN846355 | P2_Dec | Haplosporidia | V5fHapl /Sb2nHap | *Carcinus maenas* haemolymph DNA | [MN537839.1](https://www.ncbi.nlm.nih.gov/nucleotide/MN537839.1?report=genbank&log$=nucltop&blast_rank=17&RID=FSTW9HWS014) | 93 | 97.12 |
| MN846356 | P30_Feb | Haplosporidia | V5fHapl /Sb2nHap | *Carcinus maenas* haemolymph DNA | [MN537839.1](https://www.ncbi.nlm.nih.gov/nucleotide/MN537839.1?report=genbank&log$=nucltop&blast_rank=17&RID=FSTW9HWS014) | 93 | 97.62 |
| MN846357 | P24_Jun | Haplosporidia | V5fHapl /Sb2nHap | *Carcinus maenas* haemolymph DNA | [MN537839.1](https://www.ncbi.nlm.nih.gov/nucleotide/MN537839.1?report=genbank&log$=nucltop&blast_rank=17&RID=FSTW9HWS014) | 93 | 97.28 |
| MN846358 | P49_Sept | Haplosporidia | V5fHapl /Sb2nHap | *Carcinus maenas* haemolymph DNA | [MN537839.1](https://www.ncbi.nlm.nih.gov/nucleotide/MN537839.1?report=genbank&log$=nucltop&blast_rank=17&RID=FSTW9HWS014) | 93 | 97.12 |
| MN846359 | P24_Oct | Haplosporidia | V5fHapl /Sb2nHap | *Carcinus maenas* haemolymph DNA | [MN537839.1](https://www.ncbi.nlm.nih.gov/nucleotide/MN537839.1?report=genbank&log$=nucltop&blast_rank=17&RID=FSTW9HWS014) | 93 | 97.62 |
| MT334463 | Water_P1A_Nov | Haplosporidia | V5fHapl /Sb2nHap | Seawater filter (eDNA) | [KF208580.1](https://www.ncbi.nlm.nih.gov/nucleotide/KF208580.1?report=genbank&log$=nucltop&blast_rank=1&RID=FSVKTAZY016) | 89 | 95.91 |
| MT334464 | Water_P1B_Nov | Haplosporidia | V5fHapl /Sb2nHap | Seawater filter (eDNA) | [KY522823.1](https://www.ncbi.nlm.nih.gov/nucleotide/KY522823.1?report=genbank&log$=nucltop&blast_rank=1&RID=FSVM0DKP014) | 100 | 99.84 |
| MT334465 | Water_P2A_Nov | Haplosporidia | V5fHapl /Sb2nHap | Seawater filter (eDNA) | [KF208580.1](https://www.ncbi.nlm.nih.gov/nucleotide/KF208580.1?report=genbank&log$=nucltop&blast_rank=1&RID=FSVKTAZY016) | 89 | 95.91 |
| MT334466 | Water_P2B_Nov | Haplosporidia | V5fHapl /Sb2nHap | Seawater filter (eDNA) | [KY522823.1](https://www.ncbi.nlm.nih.gov/nucleotide/KY522823.1?report=genbank&log$=nucltop&blast_rank=1&RID=FSVM0DKP014) | 100 | 99.68 |
| MT334467 | Water_P3A_Nov | Haplosporidia | V5fHapl /Sb2nHap | Seawater filter (eDNA) | [KF208580.1](https://www.ncbi.nlm.nih.gov/nucleotide/KF208580.1?report=genbank&log$=nucltop&blast_rank=1&RID=FSVKTAZY016) | 89 | 95.91 |
| MT334468 | Water_P1A_Dec | Haplosporidia | V5fHapl /Sb2nHap | Seawater filter (eDNA) | [KF208581.1](https://www.ncbi.nlm.nih.gov/nucleotide/KF208581.1?report=genbank&log$=nucltop&blast_rank=1&RID=FVHU8HUV016) | 100 | 86.67 |
| MT334469 | Water_P1B_Dec | Haplosporidia | V5fHapl /Sb2nHap | Seawater filter (eDNA) | [KY522823.1](https://www.ncbi.nlm.nih.gov/nucleotide/KY522823.1?report=genbank&log$=nucltop&blast_rank=1&RID=FSVM0DKP014) | 100 | 99.84 |
| MT334470 | Water_P2A_Dec | Haplosporidia | V5fHapl /Sb2nHap | Seawater filter (eDNA) | [KF208580.1](https://www.ncbi.nlm.nih.gov/nucleotide/KF208580.1?report=genbank&log$=nucltop&blast_rank=1&RID=FSVKTAZY016) | 88 | 95.34 |
| MT334471 | Water_P3A_Dec | Haplosporidia | V5fHapl /Sb2nHap | Seawater filter (eDNA) | [KF208580.1](https://www.ncbi.nlm.nih.gov/nucleotide/KF208580.1?report=genbank&log$=nucltop&blast_rank=1&RID=FSVKTAZY016) | 89 | 95.91 |
| MT334472 | Water_P1A_Jan | Haplosporidia | V5fHapl /Sb2nHap | Seawater filter (eDNA) | [KF208580.1](https://www.ncbi.nlm.nih.gov/nucleotide/KF208580.1?report=genbank&log$=nucltop&blast_rank=1&RID=FSVKTAZY016) | 88 | 95.65 |
| MT334473 | Water_P1B_Jan | Haplosporidia | V5fHapl /Sb2nHap | Seawater filter (eDNA) | [KF208580.1](https://www.ncbi.nlm.nih.gov/nucleotide/KF208580.1?report=genbank&log$=nucltop&blast_rank=1&RID=FSVKTAZY016) | 88 | 95.34 |
| MT334474 | Water_M2Pool_Jan | Haplosporidia | V5fHapl /Sb2nHap | Seawater filter (eDNA) | [KF208580.1](https://www.ncbi.nlm.nih.gov/nucleotide/KF208580.1?report=genbank&log$=nucltop&blast_rank=1&RID=FSVKTAZY016) | 89 | 95.39 |
| MT334475 | Water_P3A_Jan | Haplosporidia | V5fHapl /Sb2nHap | Seawater filter (eDNA) | [KF208603.1](https://www.ncbi.nlm.nih.gov/nucleotide/KF208603.1?report=genbank&log$=nucltop&blast_rank=1&RID=FSXRVTA2016) | 100 | 98.88 |
| MT334476 | Water_P3B_Jan | Haplosporidia | V5fHapl /Sb2nHap | Seawater filter (eDNA) | [KF208580.1](https://www.ncbi.nlm.nih.gov/nucleotide/KF208580.1?report=genbank&log$=nucltop&blast_rank=1&RID=FSVKTAZY016) | 89 | 95.14 |
| MT334477 | Water_P2B_Feb | Haplosporidia | V5fHapl /Sb2nHap | Seawater filter (eDNA) | [KF208603.1](https://www.ncbi.nlm.nih.gov/nucleotide/KF208603.1?report=genbank&log$=nucltop&blast_rank=1&RID=FSXRVTA2016) | 100 | 99.52 |
| MT334478 | Water_P3Pool_Feb | Haplosporidia | V5fHapl /Sb2nHap | Seawater filter (eDNA) | [KF208603.1](https://www.ncbi.nlm.nih.gov/nucleotide/KF208603.1?report=genbank&log$=nucltop&blast_rank=1&RID=FSZP0UVD016) | 100 | 90.27 |
| MT334479 | Water_P1B_Mar | Haplosporidia | V5fHapl /Sb2nHap | Seawater filter (eDNA) | [MT311214.1](https://www.ncbi.nlm.nih.gov/nucleotide/MT311214.1?report=genbank&log$=nucltop&blast_rank=1&RID=FSZPFVPR014) | 100 | 99.5 |
| MT334480 | Water_P3B_Mar | Haplosporidia | V5fHapl /Sb2nHap | Seawater filter (eDNA) | [KF208580.1](https://www.ncbi.nlm.nih.gov/nucleotide/KF208580.1?report=genbank&log$=nucltop&blast_rank=1&RID=FSZPT96201R) | 89 | 95.39 |
| MT334481 | Water_P2B_Apr | Haplosporidia | V5fHapl /Sb2nHap | Seawater filter (eDNA) | [KF208580.1](https://www.ncbi.nlm.nih.gov/nucleotide/KF208580.1?report=genbank&log$=nucltop&blast_rank=1&RID=FSZPT96201R) | 89 | 95.64 |
| MT334482 | Water_P3A_Apr | Haplosporidia | V5fHapl /Sb2nHap | Seawater filter (eDNA) | [KF208580.1](https://www.ncbi.nlm.nih.gov/nucleotide/KF208580.1?report=genbank&log$=nucltop&blast_rank=1&RID=FSZPT96201R) | 89 | 95.65 |
| MT334483 | Water_P2Pool_May | Haplosporidia | V5fHapl /Sb2nHap | Seawater filter (eDNA) | [KF208580.1](https://www.ncbi.nlm.nih.gov/nucleotide/KF208580.1?report=genbank&log$=nucltop&blast_rank=1&RID=FSZPT96201R) | 86 | 95.11 |
| MT334484 | Water_P3A_May | Haplosporidia | V5fHapl /Sb2nHap | Seawater filter (eDNA) | [KF208580.1](https://www.ncbi.nlm.nih.gov/nucleotide/KF208580.1?report=genbank&log$=nucltop&blast_rank=1&RID=FSZPT96201R) | 89 | 95.37 |
| MT334485 | Water_P3B_May | Haplosporidia | V5fHapl /Sb2nHap | Seawater filter (eDNA) | [KF208580.1](https://www.ncbi.nlm.nih.gov/nucleotide/KF208580.1?report=genbank&log$=nucltop&blast_rank=1&RID=FSZPT96201R) | 89 | 95.65 |
| MT334486 | Water_P2A_Jun | Haplosporidia | V5fHapl /Sb2nHap | Seawater filter (eDNA) | [KF208580.1](https://www.ncbi.nlm.nih.gov/nucleotide/KF208580.1?report=genbank&log$=nucltop&blast_rank=1&RID=FSZPT96201R) | 89 | 95.91 |
| MT334487 | Water_P2B_Jun | Haplosporidia | V5fHapl /Sb2nHap | Seawater filter (eDNA) | [KF208580.1](https://www.ncbi.nlm.nih.gov/nucleotide/KF208580.1?report=genbank&log$=nucltop&blast_rank=1&RID=FSZPT96201R) | 89 | 95.91 |
| MT334488 | Water_P3A_Jun | Haplosporidia | V5fHapl /Sb2nHap | Seawater filter (eDNA) | [KF208596.1](https://www.ncbi.nlm.nih.gov/nucleotide/KF208596.1?report=genbank&log$=nucltop&blast_rank=1&RID=FSZTCJ2P014) | 100 | 88.53 |
| MT334489 | Water_P2B_Jul | Haplosporidia | V5fHapl /Sb2nHap | Seawater filter (eDNA) | [KY522823.1](https://www.ncbi.nlm.nih.gov/nucleotide/KY522823.1?report=genbank&log$=nucltop&blast_rank=1&RID=FSZU88N5014) | 100 | 99.36 |
| MT334490 | Water_P3B_Jul | Haplosporidia | V5fHapl /Sb2nHap | Seawater filter (eDNA) | [KY522823.1](https://www.ncbi.nlm.nih.gov/nucleotide/KY522823.1?report=genbank&log$=nucltop&blast_rank=1&RID=FSZU88N5014) | 100 | 99.36 |
| MT334491 | Water_P1A_Aug _band1 | Haplosporidia | V5fHapl /Sb2nHap | Seawater filter (eDNA) | [KF208596.1](https://www.ncbi.nlm.nih.gov/nucleotide/KF208596.1?report=genbank&log$=nucltop&blast_rank=1&RID=FT09MJDR014) | 100 | 88.53 |
| MT334492 | Water_P1A_Aug _band2 | Haplosporidia | V5fHapl /Sb2nHap | Seawater filter (eDNA) | [KY522823.1](https://www.ncbi.nlm.nih.gov/nucleotide/KY522823.1?report=genbank&log$=nucltop&blast_rank=1&RID=FT09ZJCS016) | 100 | 100 |
| MT334493 | Water_P1A_Aug _band3 | Haplosporidia | V5fHapl /Sb2nHap | Seawater filter (eDNA) | [KY522823.1](https://www.ncbi.nlm.nih.gov/nucleotide/KY522823.1?report=genbank&log$=nucltop&blast_rank=1&RID=FT09ZJCS016) | 99 | 99.64 |
| MT334494 | Water_P3A_Aug | Haplosporidia | V5fHapl /Sb2nHap | Seawater filter (eDNA) | [KY522823.1](https://www.ncbi.nlm.nih.gov/nucleotide/KY522823.1?report=genbank&log$=nucltop&blast_rank=1&RID=FT09ZJCS016) | 97 | 99.54 |
| MT334495 | Water_P3B_Aug | Haplosporidia | V5fHapl /Sb2nHap | Seawater filter (eDNA) | [KF208580.1](https://www.ncbi.nlm.nih.gov/nucleotide/KF208580.1?report=genbank&log$=nucltop&blast_rank=1&RID=FT0AUXYU01R) | 89 | 95.65 |
| MT334496 | Water_P1A_Sept | Haplosporidia | V5fHapl /Sb2nHap | Seawater filter (eDNA) | [JN368430.1](https://www.ncbi.nlm.nih.gov/nucleotide/JN368430.1?report=genbank&log$=nucltop&blast_rank=1&RID=FT0B2Y6Z01R) | 92 | 93.75 |
| MT334497 | Water_P1B_Sept | Haplosporidia | V5fHapl /Sb2nHap | Seawater filter (eDNA) | [KY522823.1](https://www.ncbi.nlm.nih.gov/nucleotide/KY522823.1?report=genbank&log$=nucltop&blast_rank=1&RID=FT0BDTG0016) | 100 | 99.2 |
| MT334498 | Water_P2A_Sept | Haplosporidia | V5fHapl /Sb2nHap | Seawater filter (eDNA) | [MK070859.1](https://www.ncbi.nlm.nih.gov/nucleotide/MK070859.1?report=genbank&log$=nucltop&blast_rank=1&RID=FT0BSK6C016) | 100 | 93.15 |
| MT334499 | Water_P2B_Sept | Haplosporidia | V5fHapl /Sb2nHap | Seawater filter (eDNA) | [KY522823.1](https://www.ncbi.nlm.nih.gov/nucleotide/KY522823.1?report=genbank&log$=nucltop&blast_rank=1&RID=FT0C53YH016) | 100 | 99.84 |
| MT334500 | Water_P3A_Sept | Haplosporidia | V5fHapl /Sb2nHap | Seawater filter (eDNA) | [KF208580.1](https://www.ncbi.nlm.nih.gov/nucleotide/KF208580.1?report=genbank&log$=nucltop&blast_rank=1&RID=FT0CHW4F014) | 89 | 95.65 |
| MT334501 | Water_P3B_Sept | Haplosporidia | V5fHapl /Sb2nHap | Seawater filter (eDNA) | [KF208596.1](https://www.ncbi.nlm.nih.gov/nucleotide/KF208596.1?report=genbank&log$=nucltop&blast_rank=1&RID=FT0CZ9CD014) | 95 | 85.52 |
| MT334502 | Water_P1A_Oct | Haplosporidia | V5fHapl /Sb2nHap | Seawater filter (eDNA) | [KF208603.1](https://www.ncbi.nlm.nih.gov/nucleotide/KF208603.1?report=genbank&log$=nucltop&blast_rank=1&RID=FT2PB618016) | 100 | 90.91 |
| MT334503 | Water_P2B_Oct | Haplosporidia | V5fHapl /Sb2nHap | Seawater filter (eDNA) | [KF208580.1](https://www.ncbi.nlm.nih.gov/nucleotide/KF208580.1?report=genbank&log$=nucltop&blast_rank=1&RID=FT2PK3PB016) | 89 | 95.14 |
| MT334504 | Water_P3A_Oct | Haplosporidia | V5fHapl /Sb2nHap | Seawater filter (eDNA) | [KF208580.1](https://www.ncbi.nlm.nih.gov/nucleotide/KF208580.1?report=genbank&log$=nucltop&blast_rank=1&RID=FT2PK3PB016) | 89 | 95.65 |
| MT334505 | Water_P3B_Oct | Haplosporidia | V5fHapl /Sb2nHap | Seawater filter (eDNA) | [KY522823.1](https://www.ncbi.nlm.nih.gov/nucleotide/KY522823.1?report=genbank&log$=nucltop&blast_rank=1&RID=FT2R7DF4014) | 100 | 99.84 |
| MT334506 | Water_P3A_Jul | Haplosporidia | V5fHapl /Sb2nHap | Seawater filter (eDNA) | [KF208603.1](https://www.ncbi.nlm.nih.gov/nucleotide/KF208603.1?report=genbank&log$=nucltop&blast_rank=1&RID=FT2RGUUD016) | 100 | 99.04 |
| MT334507 | Water_D3B_Nov | Haplosporidia | V5fHapl /Sb2nHap | Seawater filter (eDNA) | [MK070858.1](https://www.ncbi.nlm.nih.gov/nucleotide/MK070858.1?report=genbank&log$=nucltop&blast_rank=1&RID=FT2RTPWX014) | 100 | 99.03 |
| MT334508 | Water_D1B_Jan | Haplosporidia | V5fHapl /Sb2nHap | Seawater filter (eDNA) | [GU385680.1](https://www.ncbi.nlm.nih.gov/nucleotide/GU385680.1?report=genbank&log$=nucltop&blast_rank=1&RID=FT2SS8PW014) | 99 | 93.88 |
| MT334509 | Water_D1A_Feb | Haplosporidia | V5fHapl /Sb2nHap | Seawater filter (eDNA) | [KF208570.1](https://www.ncbi.nlm.nih.gov/nucleotide/KF208570.1?report=genbank&log$=nucltop&blast_rank=1&RID=FT2T2ZDF016) | 100 | 99.52 |
| MT334510 | Water_D2B_Feb | Haplosporidia | V5fHapl /Sb2nHap | Seawater filter (eDNA) | [GU385680.1](https://www.ncbi.nlm.nih.gov/nucleotide/GU385680.1?report=genbank&log$=nucltop&blast_rank=1&RID=FT2TBJSY014) | 97 | 89.93 |
| MT334511 | Water_D2A_Mar | Haplosporidia | V5fHapl /Sb2nHap | Seawater filter (eDNA) | [GU385680.1](https://www.ncbi.nlm.nih.gov/nucleotide/GU385680.1?report=genbank&log$=nucltop&blast_rank=1&RID=FT2TBJSY014) | 97 | 93.18 |
| MT334512 | Water_D1B_Apr | Haplosporidia | V5fHapl /Sb2nHap | Seawater filter (eDNA) | [DQ103863.1](https://www.ncbi.nlm.nih.gov/nucleotide/DQ103863.1?report=genbank&log$=nucltop&blast_rank=1&RID=FT2U1GHG016) | 100 | 99.81 |
| MT334513 | Water_D2B_May | Haplosporidia | V5fHapl /Sb2nHap | Seawater filter (eDNA) | [MK070858.1](https://www.ncbi.nlm.nih.gov/nucleotide/MK070858.1?report=genbank&log$=nucltop&blast_rank=1&RID=FT2UAYU5016) | 100 | 99.68 |
| MN985606 | Water_D1B_Jun | Microsporidia | CTMicrosp-G / Microsp1342r | Seawater filter (eDNA) | [KR303710.1](https://www.ncbi.nlm.nih.gov/nucleotide/KR303710.1?report=genbank&log$=nucltop&blast_rank=1&RID=FT6RG6UP01R) | 99 | 96.95 |
| MN985607 | Water_D2B_Jun | Microsporidia | CTMicrosp-G / Microsp1342r | Seawater filter (eDNA) | [FJ756177.1](https://www.ncbi.nlm.nih.gov/nucleotide/FJ756177.1?report=genbank&log$=nucltop&blast_rank=1&RID=FT6RWZR201R) | 98 | 98.84 |
| MN985608 | Water_D3B_Jun | Microsporidia | CTMicrosp-G / Microsp1342r | Seawater filter (eDNA) | [FJ756177.1](https://www.ncbi.nlm.nih.gov/nucleotide/FJ756177.1?report=genbank&log$=nucltop&blast_rank=1&RID=FT6RWZR201R) | 98 | 99.49 |
| MN985609 | Crab_D16_Aug | Microsporidia | CTMicrosp-G / Microsp1342r | *Carcinus maenas* haemolymph DNA | [MG241440.1](https://www.ncbi.nlm.nih.gov/nucleotide/MG241440.1?report=genbank&log$=nucltop&blast_rank=1&RID=FVHK1G4B014) | 100 | 83.73 |
| MN985610 | Water_P2A_Nov | Paramyxid | Para3+fN / Para2+rN | Seawater filter (eDNA) | [KX259324.1](https://www.ncbi.nlm.nih.gov/nucleotide/KX259324.1?report=genbank&log$=nucltop&blast_rank=1&RID=FT6SVH59016) | 99 | 99.5 |
| MN985611 | Water_P3A_Nov | Paramyxid | Para3+fN / Para2+rN | Seawater filter (eDNA) | [KX259324.1](https://www.ncbi.nlm.nih.gov/nucleotide/KX259324.1?report=genbank&log$=nucltop&blast_rank=1&RID=FT6SVH59016) | 100 | 100 |
| MN985612 | Water_P3B_Nov | Paramyxid | Para3+fN / Para2+rN | Seawater filter (eDNA) | [KX259324.1](https://www.ncbi.nlm.nih.gov/nucleotide/KX259324.1?report=genbank&log$=nucltop&blast_rank=1&RID=FT6SVH59016) | 87 | 91.19 |
| MN985613 | Water_P2A_Dec | Paramyxid | Para3+fN / Para2+rN | Seawater filter (eDNA) | [KX259324.1](https://www.ncbi.nlm.nih.gov/nucleotide/KX259324.1?report=genbank&log$=nucltop&blast_rank=1&RID=FT6SVH59016) | 99 | 99.75 |
| MN985614 | Water_P1A_Jan | Paramyxid | Para3+fN / Para2+rN | Seawater filter (eDNA) | [KX259324.1](https://www.ncbi.nlm.nih.gov/nucleotide/KX259324.1?report=genbank&log$=nucltop&blast_rank=1&RID=FT6SVH59016) | 100 | 99.75 |
| MN985615 | Water_P1B_Jan | Paramyxid | Para3+fN / Para2+rN | Seawater filter (eDNA) | [KX259323.1](https://www.ncbi.nlm.nih.gov/nucleotide/KX259323.1?report=genbank&log$=nucltop&blast_rank=1&RID=FT6URMCU014) | 99 | 88.91 |
| MN985616 | Water_P1B_Feb | Paramyxid | Para3+fN / Para2+rN | Seawater filter (eDNA) | [KX259324.1](https://www.ncbi.nlm.nih.gov/nucleotide/KX259324.1?report=genbank&log$=nucltop&blast_rank=1&RID=FT6V4UYV016) | 100 | 99.53 |
| MN985617 | Water_P2B_Mar | Paramyxid | Para3+fN / Para2+rN | Seawater filter (eDNA) | [KX259324.1](https://www.ncbi.nlm.nih.gov/nucleotide/KX259324.1?report=genbank&log$=nucltop&blast_rank=1&RID=FT6V4UYV016) | 100 | 99.74 |
| MN985618 | Water_P1A_Apr | Paramyxid | Para3+fN / Para2+rN | Seawater filter (eDNA) | [KX259324.1](https://www.ncbi.nlm.nih.gov/nucleotide/KX259324.1?report=genbank&log$=nucltop&blast_rank=1&RID=FT811EEW014) | 100 | 99.52 |
| MN985619 | Water_P1B_Apr | Paramyxid | Para3+fN / Para2+rN | Seawater filter (eDNA) | [KX259324.1](https://www.ncbi.nlm.nih.gov/nucleotide/KX259324.1?report=genbank&log$=nucltop&blast_rank=1&RID=FT811EEW014) | 100 | 99.5 |
| MN985620 | Water_P2A_Apr | Paramyxid | Para3+fN / Para2+rN | Seawater filter (eDNA) | [KX259324.1](https://www.ncbi.nlm.nih.gov/nucleotide/KX259324.1?report=genbank&log$=nucltop&blast_rank=1&RID=FT811EEW014) | 100 | 99.75 |
| MN985621 | Water_P2B_Apr | Paramyxid | Para3+fN / Para2+rN | Seawater filter (eDNA) | [KX259324.1](https://www.ncbi.nlm.nih.gov/nucleotide/KX259324.1?report=genbank&log$=nucltop&blast_rank=1&RID=FT811EEW014) | 99 | 100 |
| MN985622 | Water_P3B_Apr | Paramyxid | Para3+fN / Para2+rN | Seawater filter (eDNA) | [KX259324.1](https://www.ncbi.nlm.nih.gov/nucleotide/KX259324.1?report=genbank&log$=nucltop&blast_rank=1&RID=FT811EEW014) | 99 | 99.75 |
| MN985623 | Water_P2A_May | Paramyxid | Para3+fN / Para2+rN | Seawater filter (eDNA) | [KX259324.1](https://www.ncbi.nlm.nih.gov/nucleotide/KX259324.1?report=genbank&log$=nucltop&blast_rank=1&RID=FT811EEW014) | 100 | 99.05 |
| MN985624 | Water_P2B_May | Paramyxid | Para3+fN / Para2+rN | Seawater filter (eDNA) | [KX259324.1](https://www.ncbi.nlm.nih.gov/nucleotide/KX259324.1?report=genbank&log$=nucltop&blast_rank=1&RID=FT811EEW014) | 100 | 100 |
| MN985625 | Water_P2B_May(2) | Paramyxid | Para3+fN / Para2+rN | Seawater filter (eDNA) | [KX259324.1](https://www.ncbi.nlm.nih.gov/nucleotide/KX259324.1?report=genbank&log$=nucltop&blast_rank=1&RID=FT811EEW014) | 100 | 99.5 |
| MN985626 | Water_P3A_May | Paramyxid | Para3+fN / Para2+rN | Seawater filter (eDNA) | [KX259324.1](https://www.ncbi.nlm.nih.gov/nucleotide/KX259324.1?report=genbank&log$=nucltop&blast_rank=1&RID=FT811EEW014) | 100 | 100 |
| MN985627 | Water_P1B_Jun | Paramyxid | Para3+fN / Para2+rN | Seawater filter (eDNA) | [KX259327.1](https://www.ncbi.nlm.nih.gov/nucleotide/KX259327.1?report=genbank&log$=nucltop&blast_rank=1&RID=FT854AXX016) | 100 | 93.36 |
| MN985628 | Water_P2B_Jun | Paramyxid | Para3+fN / Para2+rN | Seawater filter (eDNA) | [KX259327.1](https://www.ncbi.nlm.nih.gov/nucleotide/KX259327.1?report=genbank&log$=nucltop&blast_rank=1&RID=FT854AXX016) | 99 | 100 |
| MN985629 | Water_P3B_Jun | Paramyxid | Para3+fN / Para2+rN | Seawater filter (eDNA) | [KX259324.1](https://www.ncbi.nlm.nih.gov/nucleotide/KX259324.1?report=genbank&log$=nucltop&blast_rank=1&RID=FT85ZXG4016) | 99 | 99.75 |
| MN985630 | Water_P1A_Jul | Paramyxid | Para3+fN / Para2+rN | Seawater filter (eDNA) | [KX259324.1](https://www.ncbi.nlm.nih.gov/nucleotide/KX259324.1?report=genbank&log$=nucltop&blast_rank=1&RID=FT8RUXAG016) | 99 | 99.75 |
| MN985631 | Water_P1B_Jul | Paramyxid | Para3+fN / Para2+rN | Seawater filter (eDNA) | [MH304633.1](https://www.ncbi.nlm.nih.gov/nucleotide/MH304633.1?report=genbank&log$=nucltop&blast_rank=1&RID=FT8S2YYH014) | 94 | 96.37 |
| MN985632 | Water_P1B_Jul(2) | Paramyxid | Para3+fN / Para2+rN | Seawater filter (eDNA) | [MH304633.1](https://www.ncbi.nlm.nih.gov/nucleotide/MH304633.1?report=genbank&log$=nucltop&blast_rank=1&RID=FT8SB18C016) | 100 | 99.07 |
| MN985633 | Water_P1A_Aug | Paramyxid | Para3+fN / Para2+rN | Seawater filter (eDNA) | [KX259324.1](https://www.ncbi.nlm.nih.gov/nucleotide/KX259324.1?report=genbank&log$=nucltop&blast_rank=1&RID=FT8SP0FF014) | 100 | 99.48 |
| MN985634 | Water_P2B_Aug | Paramyxid | Para3+fN / Para2+rN | Seawater filter (eDNA) | [KX259324.1](https://www.ncbi.nlm.nih.gov/nucleotide/KX259324.1?report=genbank&log$=nucltop&blast_rank=1&RID=FT8SP0FF014) | 100 | 99.48 |
| MN985635 | Water_P3A_Aug | Paramyxid | Para3+fN / Para2+rN | Seawater filter (eDNA) | [KX259324.1](https://www.ncbi.nlm.nih.gov/nucleotide/KX259324.1?report=genbank&log$=nucltop&blast_rank=1&RID=FT8SP0FF014) | 99 | 99.75 |
| MN985636 | Water_P1B_Sept_1 | Paramyxid | Para3+fN / Para2+rN | Seawater filter (eDNA) | [KX259324.1](https://www.ncbi.nlm.nih.gov/nucleotide/KX259324.1?report=genbank&log$=nucltop&blast_rank=1&RID=FT8SP0FF014) | 99 | 99.75 |
| MN985637 | Water_P1B_Sept_2 | Paramyxid | Para3+fN / Para2+rN | Seawater filter (eDNA) | [MH304633.1](https://www.ncbi.nlm.nih.gov/nucleotide/MH304633.1?report=genbank&log$=nucltop&blast_rank=1&RID=FT8UYWSS014) | 100 | 99.3 |
| MN985638 | Water_P2B_Sept | Paramyxid | Para3+fN / Para2+rN | Seawater filter (eDNA) | [KX259324.1](https://www.ncbi.nlm.nih.gov/nucleotide/KX259324.1?report=genbank&log$=nucltop&blast_rank=1&RID=FT8VJKKJ014) | 100 | 100 |
| MN985639 | Water_P3A_Sept | Paramyxid | Para3+fN / Para2+rN | Seawater filter (eDNA) | [KX259324.1](https://www.ncbi.nlm.nih.gov/nucleotide/KX259324.1?report=genbank&log$=nucltop&blast_rank=1&RID=FT8VJKKJ014) | 99 | 100 |
| MN985640 | Water_P3B_Sept | Paramyxid | Para3+fN / Para2+rN | Seawater filter (eDNA) | [KX259324.1](https://www.ncbi.nlm.nih.gov/nucleotide/KX259324.1?report=genbank&log$=nucltop&blast_rank=1&RID=FT8VJKKJ014) | 100 | 99.5 |
| MN985641 | Water_P1A_Oct_1 | Paramyxid | Para3+fN / Para2+rN | Seawater filter (eDNA) | [MH304633.1](https://www.ncbi.nlm.nih.gov/nucleotide/MH304633.1?report=genbank&log$=nucltop&blast_rank=1&RID=FT8XNWVS014) | 94 | 91.96 |
| MN985642 | Water_P1A_Oct_2 | Paramyxid | Para3+fN / Para2+rN | Seawater filter (eDNA) | [MH304633.1](https://www.ncbi.nlm.nih.gov/nucleotide/MH304633.1?report=genbank&log$=nucltop&blast_rank=1&RID=FTAZK0TY01R) | 100 | 98.84 |
| MN985643 | Water_P1B_Oct | Paramyxid | Para3+fN / Para2+rN | Seawater filter (eDNA) | [MH304633.1](https://www.ncbi.nlm.nih.gov/nucleotide/MH304633.1?report=genbank&log$=nucltop&blast_rank=1&RID=FTAZK0TY01R) | 100 | 99.07 |
| MN985644 | Water_P3A_Oct | Paramyxid | Para3+fN / Para2+rN | Seawater filter (eDNA) | [MH304633.1](https://www.ncbi.nlm.nih.gov/nucleotide/MH304633.1?report=genbank&log$=nucltop&blast_rank=1&RID=FTAZK0TY01R) | 91 | 91.63 |
| MT000071 | Water_D3A_Nov | Mikrocytid | mik868F / mik1340R | Seawater filter (eDNA) | [KF297353.1](https://www.ncbi.nlm.nih.gov/nucleotide/KF297353.1?report=genbank&log$=nucltop&blast_rank=1&RID=FTB1VCYP016) | 100 | 100 |
| MT000072 | Water_P2A_Nov | Mikrocytid | mik868F / mik1340R | Seawater filter (eDNA) | [KJ150292.1](https://www.ncbi.nlm.nih.gov/nucleotide/KJ150292.1?report=genbank&log$=nucltop&blast_rank=1&RID=FTB25ZEB016) | 100 | 99.56 |
| MT000073 | Water_P3A_Nov | Mikrocytid | mik868F / mik1340R | Seawater filter (eDNA) | [KF548050.1](https://www.ncbi.nlm.nih.gov/nucleotide/KF548050.1?report=genbank&log$=nucltop&blast_rank=1&RID=FTB2F3R5014) | 83 | 82.89 |
| MT000074 | Water_P1A_Dec | Mikrocytid | mik868F / mik1340R | Seawater filter (eDNA) | [KF548050.1](https://www.ncbi.nlm.nih.gov/nucleotide/KF548050.1?report=genbank&log$=nucltop&blast_rank=1&RID=FTB2F3R5014) | 100 | 87.53 |
| MT000075 | Water_D1A_Jan | Mikrocytid | mik868F / mik1340R | Seawater filter (eDNA) | [KF297353.1](https://www.ncbi.nlm.nih.gov/nucleotide/KF297353.1?report=genbank&log$=nucltop&blast_rank=1&RID=FTB31U8J016) | 100 | 100 |
| MT000076 | Water_P3B_Jan | Mikrocytid | mik868F / mik1340R | Seawater filter (eDNA) | [KF548050.1](https://www.ncbi.nlm.nih.gov/nucleotide/KF548050.1?report=genbank&log$=nucltop&blast_rank=1&RID=FTB398ZJ016) | 100 | 87.76 |
| MT000077 | Water_D1A_Feb | Mikrocytid | mik868F / mik1340R | Seawater filter (eDNA) | [KF297353.1](https://www.ncbi.nlm.nih.gov/nucleotide/KF297353.1?report=genbank&log$=nucltop&blast_rank=1&RID=FTB3M2G3016) | 100 | 100 |
| MT000078 | Water_D2A_Feb | Mikrocytid | mik868F / mik1340R | Seawater filter (eDNA) | [KF297353.1](https://www.ncbi.nlm.nih.gov/nucleotide/KF297353.1?report=genbank&log$=nucltop&blast_rank=1&RID=FTB3M2G3016) | 100 | 100 |
| MT000079 | Water_P3A_Feb | Mikrocytid | mik868F / mik1340R | Seawater filter (eDNA) | [KF548050.1](https://www.ncbi.nlm.nih.gov/nucleotide/KF548050.1?report=genbank&log$=nucltop&blast_rank=1&RID=FTB4ASSF014) | 100 | 87.73 |
| MT000080 | Water_D1B_Jun | Mikrocytid | mik868F / mik1340R | Seawater filter (eDNA) | [KF297353.1](https://www.ncbi.nlm.nih.gov/nucleotide/KF297353.1?report=genbank&log$=nucltop&blast_rank=1&RID=FTBG6D2N016) | 100 | 99.58 |
| MT000081 | Water_D2B_Jun | Mikrocytid | mik868F / mik1340R | Seawater filter (eDNA) | [KF297353.1](https://www.ncbi.nlm.nih.gov/nucleotide/KF297353.1?report=genbank&log$=nucltop&blast_rank=1&RID=FTBG6D2N016) | 100 | 100 |
| MT000082 | Water_D3B_Jun | Mikrocytid | mik868F / mik1340R | Seawater filter (eDNA) | [KF297353.1](https://www.ncbi.nlm.nih.gov/nucleotide/KF297353.1?report=genbank&log$=nucltop&blast_rank=1&RID=FTBG6D2N016) | 100 | 99.57 |
| MT000083 | Water_D1A_Jul | Mikrocytid | mik868F / mik1340R | Seawater filter (eDNA) | [KF297353.1](https://www.ncbi.nlm.nih.gov/nucleotide/KF297353.1?report=genbank&log$=nucltop&blast_rank=1&RID=FTBG6D2N016) | 100 | 98.72 |
| MT000084 | Water_D2A_Jul | Mikrocytid | mik868F / mik1340R | Seawater filter (eDNA) | [KF297353.1](https://www.ncbi.nlm.nih.gov/nucleotide/KF297353.1?report=genbank&log$=nucltop&blast_rank=1&RID=FTBG6D2N016) | 100 | 100 |
| MT000085 | Water_D3A_Jul | Mikrocytid | mik868F / mik1340R | Seawater filter (eDNA) | [KF297353.1](https://www.ncbi.nlm.nih.gov/nucleotide/KF297353.1?report=genbank&log$=nucltop&blast_rank=1&RID=FTBG6D2N016) | 100 | 100 |
| MT000086 | Water_D3B_Jul | Mikrocytid | mik868F / mik1340R | Seawater filter (eDNA) | [KF297353.1](https://www.ncbi.nlm.nih.gov/nucleotide/KF297353.1?report=genbank&log$=nucltop&blast_rank=1&RID=FTBG6D2N016) | 100 | 100 |
| MT000087 | Water_P3A_Aug | Mikrocytid | mik868F / mik1340R | Seawater filter (eDNA) | [KF548050.1](https://www.ncbi.nlm.nih.gov/nucleotide/KF548050.1?report=genbank&log$=nucltop&blast_rank=1&RID=FTBJSX1M01R) | 100 | 87.73 |
| MT000088 | Water_P1A_Sep | Mikrocytid | mik868F / mik1340R | Seawater filter (eDNA) | [KF548050.1](https://www.ncbi.nlm.nih.gov/nucleotide/KF548050.1?report=genbank&log$=nucltop&blast_rank=1&RID=FTBJSX1M01R) | 100 | 87.73 |
| MT000089 | Water_P2A_Sep | Mikrocytid | mik868F / mik1340R | Seawater filter (eDNA) | [KF548050.1](https://www.ncbi.nlm.nih.gov/nucleotide/KF548050.1?report=genbank&log$=nucltop&blast_rank=1&RID=FTBJSX1M01R) | 100 | 87.73 |
| MT000090 | Water_P3A_Sep | Mikrocytid | mik868F / mik1340R | Seawater filter (eDNA) | [KF548050.1](https://www.ncbi.nlm.nih.gov/nucleotide/KF548050.1?report=genbank&log$=nucltop&blast_rank=1&RID=FTBJSX1M01R) | 100 | 87.53 |
| MT000091 | Water_D1A_Oct | Mikrocytid | mik868F / mik1340R | Seawater filter (eDNA) | [KF297353.1](https://www.ncbi.nlm.nih.gov/nucleotide/KF297353.1?report=genbank&log$=nucltop&blast_rank=1&RID=FTBM3D7T016) | 100 | 99.79 |
| MT000092 | Water_D3A_Oct | Mikrocytid | mik868F / mik1340R | Seawater filter (eDNA) | [KF297353.1](https://www.ncbi.nlm.nih.gov/nucleotide/KF297353.1?report=genbank&log$=nucltop&blast_rank=1&RID=FTC6RY8D014) | 100 | 100 |
| MT000093 | Water_P1A_Oct | Mikrocytid | mik868F / mik1340R | Seawater filter (eDNA) | [KF548050.1](https://www.ncbi.nlm.nih.gov/nucleotide/KF548050.1?report=genbank&log$=nucltop&blast_rank=1&RID=FTC264NH014) | 100 | 87.73 |
| MT000094 | Water_P2A_Oct | Mikrocytid | mik868F / mik1340R | Seawater filter (eDNA) | [KP164508.1](https://www.ncbi.nlm.nih.gov/nucleotide/KP164508.1?report=genbank&log$=nucltop&blast_rank=1&RID=FTC2G38201R) | 94 | 88.14 |
| MT000095 | Water_P3A_Oct | Mikrocytid | mik868F / mik1340R | Seawater filter (eDNA) | [KJ150292.1](https://www.ncbi.nlm.nih.gov/nucleotide/KJ150292.1?report=genbank&log$=nucltop&blast_rank=1&RID=FTC2RM6T01R) | 100 | 98.91 |
| MT000096 | Water_P1B_Oct | Mikrocytid | mik868F / mik1340R | Seawater filter (eDNA) | [KJ150292.1](https://www.ncbi.nlm.nih.gov/nucleotide/KJ150292.1?report=genbank&log$=nucltop&blast_rank=1&RID=FTC2RM6T01R) | 100 | 99.56 |
| MT000097 | Water_P3B_Oct | Mikrocytid | mik868F / mik1340R | Seawater filter (eDNA) | [KJ150292.1](https://www.ncbi.nlm.nih.gov/nucleotide/KJ150292.1?report=genbank&log$=nucltop&blast_rank=1&RID=FTC2RM6T01R) | 93 | 92.31 |
| MT000098 | Water_D1B_Nov | Mikrocytid | mik868F / mik1340R | Seawater filter (eDNA) | [KF297353.1](https://www.ncbi.nlm.nih.gov/nucleotide/KF297353.1?report=genbank&log$=nucltop&blast_rank=1&RID=FTC3HYNE01R) | 100 | 99.15 |
| MT000100 | Crab_P5_Dec | Fungal species | ITS1F / ITS2 | *Carcinus maenas* haemolymph DNA | [HM119586.1](https://www.ncbi.nlm.nih.gov/nucleotide/HM119586.1?report=genbank&log$=nucltop&blast_rank=1&RID=FTC3UK6G016) | 100 | 88.55 |
| MT000101 | Crab_P50_May | Fungal species | ITS1F / ITS2 | *Carcinus maenas* haemolymph DNA | [HM119586.1](https://www.ncbi.nlm.nih.gov/nucleotide/HM119586.1?report=genbank&log$=nucltop&blast_rank=1&RID=FTC4260W014) | 100 | 87.88 |
| MT000102 | Crab_P21_Aug | Fungal species | ITS1F / ITS2 | *Carcinus maenas* haemolymph DNA | [HM119586.1](https://www.ncbi.nlm.nih.gov/nucleotide/HM119586.1?report=genbank&log$=nucltop&blast_rank=1&RID=FTC4260W014) | 100 | 87.88 |
| MT000103 | Crab_P28_Nov | Fungal species | ITS1F / ITS2 | *Carcinus maenas* haemolymph DNA | [JQ038334.1](https://www.ncbi.nlm.nih.gov/nucleotide/JQ038334.1?report=genbank&log$=nucltop&blast_rank=1&RID=FTC4TNBZ014) | 100 | 86.18 |
| MT000104 | Water_D3A_Mar | Fungal species | ITS1F / ITS2 | Seawater filter (eDNA) | [KY115002.1](https://www.ncbi.nlm.nih.gov/nucleotide/KY115002.1?report=genbank&log$=nucltop&blast_rank=1&RID=FTC52UN7016) | 28 | 96.25 |
| MT000105 | Water_D1A_Apr | Fungal species | ITS1F / ITS2 | Seawater filter (eDNA) | [MH625678.1](https://www.ncbi.nlm.nih.gov/nucleotide/MH625678.1?report=genbank&log$=nucltop&blast_rank=1&RID=FTC5C835016) | 22 | 95.59 |
| MT000106 | Water_D1A_May | Fungal species | ITS1F / ITS2 | Seawater filter (eDNA) | [KY115002.1](https://www.ncbi.nlm.nih.gov/nucleotide/KY115002.1?report=genbank&log$=nucltop&blast_rank=1&RID=FTC6612D016) | 32 | 89.13 |
| MT000107 | Water_D2A_Aug | Fungal species | ITS1F / ITS2 | Seawater filter (eDNA) | [MH625594.1](https://www.ncbi.nlm.nih.gov/nucleotide/MH625594.1?report=genbank&log$=nucltop&blast_rank=1&RID=FTCHBBCP01R) | 100 | 76.65 |
| SAMN14133753 | Crab_D15_Jul | *Vibrio* sp. | 567F / 680R | *Carcinus maenas* haemolymph DNA | [MT510186.1](https://www.ncbi.nlm.nih.gov/nucleotide/MT510186.1?report=genbank&log$=nucltop&blast_rank=1&RID=FTCHJ8UN01R) | 100 | 100 |
| SAMN14133754 | Crab_P6_Sept | *Vibrio* sp. | 567F / 680R | *Carcinus maenas* haemolymph DNA | [MT510186.1](https://www.ncbi.nlm.nih.gov/nucleotide/MT510186.1?report=genbank&log$=nucltop&blast_rank=1&RID=FTCHJ8UN01R) | 100 | 98.63 |
| SAMN14133755 | Crab_D49_Oct | *Vibrio* sp. | 567F / 680R | *Carcinus maenas* haemolymph DNA | *NONE* |  |  |
| SAMN14133756 | Crab_D18_Aug | *Vibrio* sp. | 567F / 680R | *Carcinus maenas* haemolymph DNA | [MT634723.1](https://www.ncbi.nlm.nih.gov/nucleotide/MT634723.1?report=genbank&log$=nucltop&blast_rank=1&RID=FTCJ8VMK01R) | 95 | 100 |
| SAMN14133757 | Crab_D16_Aug | *Vibrio* sp. | 567F / 680R | *Carcinus maenas* haemolymph DNA | [MT634723.1](https://www.ncbi.nlm.nih.gov/nucleotide/MT634723.1?report=genbank&log$=nucltop&blast_rank=1&RID=FTCJ8VMK01R) | 70 | 97.96 |
| SAMN14133758 | Water_P1B_Jul | *Vibrio* sp. | 567F / 680R | Seawater filter (eDNA) | [KF941973.1](https://www.ncbi.nlm.nih.gov/nucleotide/KF941973.1?report=genbank&log$=nucltop&blast_rank=1&RID=FTCJPN9Y01R) | 98 | 94.52 |
| SAMN14133759 | Water_P1B_Aug | *Vibrio* sp. | 567F / 680R | Seawater filter (eDNA) | [MT510186.1](https://www.ncbi.nlm.nih.gov/nucleotide/MT510186.1?report=genbank&log$=nucltop&blast_rank=1&RID=FTCJYD7801R) | 100 | 98.63 |
| SAMN14133760 | Water_P1B_Sept | *Vibrio* sp. | 567F / 680R | Seawater filter (eDNA) | [LN871542.1](https://www.ncbi.nlm.nih.gov/nucleotide/LN871542.1?report=genbank&log$=nucltop&blast_rank=1&RID=FTCK7HNS01R) | 88 | 97.01 |
| SAMN14133761 | Water_P1B_Oct | *Vibrio* sp. | 567F / 680R | Seawater filter (eDNA) | [HE584789.1](https://www.ncbi.nlm.nih.gov/nucleotide/HE584789.1?report=genbank&log$=nucltop&blast_rank=1&RID=FTCKGXMY014) | 96 | 89.23 |
| SAMN14133762 | Water_D1B_Jul | *Vibrio* sp. | 567F / 680R | Seawater filter (eDNA) | [MN023424.1](https://www.ncbi.nlm.nih.gov/nucleotide/MN023424.1?report=genbank&log$=nucltop&blast_rank=1&RID=FTCKWWGF014) | 100 | 97.3 |
| SAMN14133763 | Water_D1B_Aug | *Vibrio* sp. | 567F / 680R | Seawater filter (eDNA) | [MT510186.1](https://www.ncbi.nlm.nih.gov/nucleotide/MT510186.1?report=genbank&log$=nucltop&blast_rank=1&RID=FTCM63PB016) | 100 | 98.63 |
| SAMN14133764 | Water_D1B_Sept | *Vibrio* sp. | 567F / 680R | Seawater filter (eDNA) | [KY282191.1](https://www.ncbi.nlm.nih.gov/nucleotide/KY282191.1?report=genbank&log$=nucltop&blast_rank=1&RID=FTCMFDER01R) | 72 | 100 |
| SAMN14133765 | Water_D1B_Oct | *Vibrio* sp. | 567F / 680R | Seawater filter (eDNA) | [KF994032.1](https://www.ncbi.nlm.nih.gov/nucleotide/KF994032.1?report=genbank&log$=nucltop&blast_rank=1&RID=FTCMPY6T01R) | 98 | 94.44 |
