# Supplementary material for: Hematodinium sp. infection does not drive collateral disease contraction in a crustacean host
Source: eLife. 2022 Feb 18;11:e70356. doi: 10.7554/eLife.70356 (PMC8856654; doi:10.7554/eLife.70356)
Supplement: Transparent reporting form [file elife-70356-transrepform1.docx]

***eLife’s* transparent reporting form**

We encourage authors to provide detailed information *within their submission* to facilitate the interpretation and replication of experiments. Authors can upload supporting documentation to indicate the use of appropriate reporting guidelines for health-related research (see [EQUATOR Network](http://www.equator-network.org/%20)), life science research (see the [BioSharing Information Resource](https://biosharing.org/)), or the [ARRIVE guidelines](http://www.plosbiology.org/article/info:doi/10.1371/journal.pbio.1000412) for reporting work involving animal research. Where applicable, authors should refer to any relevant reporting standards documents in this form.

If you have any questions, please consult our Journal Policies and/or contact us: [editorial@elifesciences.org](mailto:editorial@elifesciences.org).

**Sample-size estimation**

- You should state whether an appropriate sample size was computed when the study was being designed
- You should state the statistical method of sample size computation and any required assumptions
- If no explicit power analysis was used, you should describe how you decided what sample (replicate) size (number) to use

Please outline where this information can be found within the submission (e.g., sections or figure legends), or explain why this information doesn’t apply to your submission:

The initial sample size and corresponding power calculation for the *Hematodinium* survey was calculated based on previously published works (Davies et al., 2019, <https://link.springer.com/article/10.1186/s13071-019-3727-x>). Using an alpha value of 0.05 and desired power above 80%, a minimum of 38 (1-sided test) up to 48 (2-sided test) based on an *a priori* prediction of 15% parasite prevalence in line with the results from Smith et al. (2015; Parasitology) in another crab species at a similar location, *C. pagurus*.

**Replicates**

- You should report how often each experiment was performed
- You should include a definition of biological versus technical replication
- The data obtained should be provided and sufficient information should be provided to indicate the number of independent biological and/or technical replicates
- If you encountered any outliers, you should describe how these were handled
- Criteria for exclusion/inclusion of data should be clearly stated
- High-throughput sequence data should be uploaded before submission, with a private link for reviewers provided (these are available from both GEO and ArrayExpress)

Please outline where this information can be found within the submission (e.g., sections or figure legends), or explain why this information doesn’t apply to your submission:

From above, ~50 crabs per site per month were screened initially for the presence/absence of parasite (n = 1,191 in total). An additional 58 crabs were processed in July 2021 and 7 in October 2021 as part of the revision efforts.

PCR based detection revealed a prevalence of ~14% across both sites, equating to 162 animals in total. In the current study, we size/sex/location-matched the 162-Hematodinium positive crabs with another 162 Hematodinium-negative animals (confirmed via PCR, haematology, and multi-tissue histology) that were collected as part of the 1,191 crabs from the year-long survey. Each of these animals represents a biological replicate. For the revised text, we ran models with both the total 162- parasitized crabs (PCR-positive), and further models with the parasitized crabs that showed clinical levels of infection (n = 111), i.e., those positive via PCR, liquid and tissue screening (see Supplementary information).

All sequence data from PCR-generated amplicons and Sanger’s chemistry have been deposited in GenBank and are listed in supporting information (see Supplementary information). All parasite prevalence data has been provided, and a table formatted for generating the nMDS plots (see Supplementary information) has been included as supporting information.

Full binomial logistic regression model outputs have been provided in the supporting information, and the reduced final models have been included in our main text (Table 1).

**Statistical reporting**

- Statistical analysis methods should be described and justified
- Raw data should be presented in figures whenever informative to do so (typically when N per group is less than 10)
- For each experiment, you should identify the statistical tests used, exact values of N, definitions of center, methods of multiple test correction, and dispersion and precision measures (e.g., mean, median, SD, SEM, confidence intervals; and, for the major substantive results, a measure of effect size (e.g., Pearson's r, Cohen's d)
- Report exact p-values wherever possible alongside the summary statistics and 95% confidence intervals. These should be reported for all key questions and not only when the p-value is less than 0.05.

Please outline where this information can be found within the submission (e.g., sections or figure legends), or explain why this information doesn’t apply to your submission:

Our overall sample sizes for multi-parasite inspection, 162-Hematodinium-positive and 162 Hematodinium negative crabs (n = 324, out of 1,191 crabs collected) is stated clearly in several sections. An additional 58 crabs were processed as part of the revision – this is stated explicitly in the same section. All n- values are displayed in the corresponding figure and/or results text, e.g., Figure 2. There is no instance where an n = 10 or fewer. Each of the statistical tests used have been listed clearly in the detailed ‘Statistical Analyses’ section of our Methods, and in the results text. Precise P-values have been reported in the text, no exceptions. In figures, sometimes statistical differences are indicated with a symbol (asterisk), but these are reported in full in the main text.

(For large datasets, or papers with a very large number of statistical tests, you may upload a single table file with tests, Ns, etc., with reference to sections in the manuscript.)

**Group allocation**

- Indicate how samples were allocated into experimental groups (in the case of clinical studies, please specify allocation to treatment method); if randomization was used, please also state if restricted randomization was applied
- Indicate if masking was used during group allocation, data collection and/or data analysis

Please outline where this information can be found within the submission (e.g., sections or figure legends), or explain why this information doesn’t apply to your submission:

All crabs collected in our survey – as stated above – were tested for the parasite Hematodinium, animals that were positive via PCR (162) where then matched to crabs that were parasite-free (162), but shared location, carapace width and sex. Randomization was not necessary as all animals formed part of the initial screen.

**Additional data files (“source data”)**

- We encourage you to upload relevant additional data files, such as numerical data that are represented as a graph in a figure, or as a summary table
- Where provided, these should be in the most useful format, and they can be uploaded as “Source data” files linked to a main figure or table
- Include model definition files including the full list of parameters used
- Include code used for data analysis (e.g., R, MatLab)
- Avoid stating that data files are “available upon request”

Please indicate the figures or tables for which source data files have been provided:

All data is available immediately, not upon request.

Source files/code, and raw data underlying the figures presented have been included in full.

R-scripts for all Binomial Logistic Regression Models (Source code 1), the revised models (Source code 2) and other data analyses have been supplied in full.

Images of histological assessment of the 324 crabs are representative of the macro/micro parasites observed.
